# Towards Tartaric-Acid-Derived Asymmetric Organocatalysts

**DOI:** 10.1002/ejoc.201201675

**Published:** 2013-06-03

**Authors:** Katharina Gratzer, Guddeangadi N Gururaja, Mario Waser

**Affiliations:** a Institute of Organic Chemistry, Johannes Kepler University LinzAltenbergerstraße 69, 4040 Linz, Austria, Fax: +43-732-2468-8747http://www.orc.jku.at/mwaser

**Keywords:** TADDOLs, Chiral pool, H-bonding catalysis, Phase-transfer catalysis, Chiral Lewis bases, Asymmetric catalysis

## Abstract

Tartaric acid is one of the most prominent naturally occurring chiral compounds. Whereas its application in the production of chiral ligands for metal-catalysed reactions has been exhaustively investigated, its potential to provide new organocatalysts has been less extensively explored. Nevertheless, some impressive results, such as the use of TADDOLs as chiral H-bonding catalysts or of tartrate-derived asymmetric quaternary ammonium salt catalysts, have been reported over the last decade. The goal of this article is to provide a representative overview of the potential and the limitations of tartaric acid or TADDOLs in the creation of new organocatalysts and to highlight some of the most spectacular applications of these catalysts, as well as to summarize case studies in which other classes of chiral backbones were better suited.

## Introduction

The ability to control the three-dimensional structure of molecular architecture is one of the primary targets in synthetic organic chemistry. The field of asymmetric synthesis has made spectacular progress over the last few decades. Of the various ways of creating enantiomerically enriched products, catalytic methods are considered the most appealing. Besides enzymatic and metal-catalysed asymmetric transformations, the use of organocatalysts has proved to have enormous potential for the catalysis of stereoselective reactions.^1^

**Figure 1:**
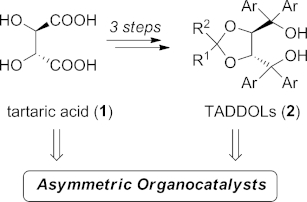
Tartaric acid (1) and TADDOLs 2 as easily available starting materials for production of asymmetric organocatalysts.

Of the easily available natural chiral sources, tartaric acid (**1**, Figure 1) has achieved a privileged position, due especially to the fact that both enantiomers are easily available. Compound **1** has thus become a valuable and cheap source of primary chiral information for asymmetric catalysis. Over the years unmodified **1** has been employed in a variety of different applications.^2^ In addition, **1** has also emerged as the foundation of a privileged class of starting materials, being used to obtain systematically fine-tuned derivatives such as tetraaryl-2,2-dimethyl-1,3-dioxolan-4,5-dimethanols **2** (TADDOLs) and analogous compounds, which have found numerous and widespread applications as chiral ligands in asymmetric metal-catalysed transformations.^3^,^4^ Surprisingly, though, whereas ligands derived from **1** or **2** are omnipresent in (transition) metal catalysis, their use to provide chiral organocatalysts has so far been less exhaustively investigated. Nevertheless, some inspiring and impressive results and applications have been reported over the last decade, and it is the target of this article to provide the interested reader with a representative overview of the potential and the limitations of tartaric acid or TADDOLs in providing new organocatalysts.

## Chiral Brønsted Acids and H-Bonding Donors

In view of the high potential both of strong Brønsted acids and of weaker hydrogen-bond donors as (chiral or achiral) small-molecule catalysts to activate (predominantly) electrophiles to facilitate different transformations, a variety of catalysts have been introduced successfully over the last few years.^5^ Although most chiral catalysts developed so far are mainly based on the binaphthyl backbone or the *trans*-cyclohexane moiety, syntheses and applications of Brønsted acidic catalysts or H-bonding catalysts derived from tartaric acid or TADDOLs have been thoroughly investigated over the last decade.

### Carboxylic Acid Derivatives

**Scheme 1:**
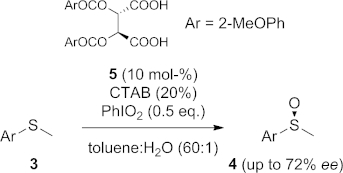
Asymmetric sulfide oxidation catalysed by tartaric acid derivative 5.

In 1999, Kita et al. reported the asymmetric hypervalent-iodine-mediated oxidation of sulfides **3** to sulfoxides **4** (Scheme 1).^6^ Carrying out these reactions in a reversed micellar system [toluene/H_2_O solvent mixture with cetyltrimethylammonium bromide (CTAB)], they identified the dibenzoyl tartaric acid derivative **5** as the most active chiral source, obtaining sulfones **4** in up to 72 % *ee*.^6^

Although this early example illustrated the potential of modified tartaric acid derivatives in asymmetric catalysis, the use of such modified dicarboxylic acids (apart from the use of **1** itself for different applications^2^) has attracted only limited interest over recent years.

### Diols

**Scheme 2:**
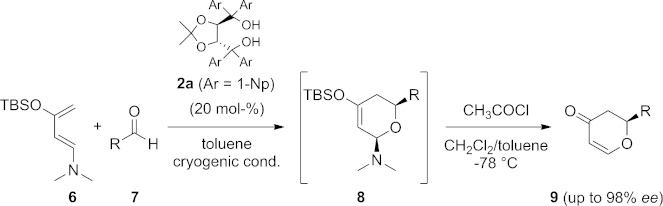
Rawal’s TADDOL-catalysed hetero-Diels–Alder reactions.

In contrast to the use of carboxylic acid derivatives based on **1**, the use of TADDOLs **2** as chiral H-bonding catalysts has emerged as a powerful and versatile tool in a variety of different applications. The seminal report in this field was published in 2003 by Rawal et al., who reported the use of TADDOLs as chiral H-bonding catalysts to facilitate highly enantioselective hetero-Diels–Alder reactions (Scheme 2).^7^ On treatment of diene **6** with different aldehydes **7** in the presence of TADDOL catalyst **2a** the hetero-Diels–Alder products **8** were formed first and were then directly further converted into almost enantiopure dihydropyrones **9**.

**Scheme 3:**
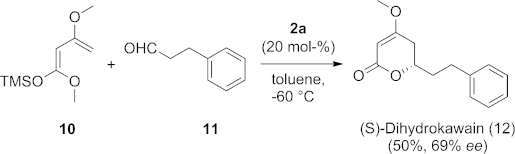
TADDOL-catalysed hetero-Diels–Alder reaction in the synthesis of (*S*)-dihydrokawain (12).

Not surprisingly, this impressive report soon spurred further investigations into broader application and also found its way into the repertoire of organic chemists interested in natural product synthesis.^8^ In 2004, Ding et al. used catalyst **2a** to access (*S*)-dihydrokawain (**12**, Scheme 3) in a single step from Brassard’s diene **10** and phenylpropanal (**11**).^8^ Although yield and enantioselectivity were only modest (69 % *ee*), this report underscores the considerable potential of hydrogen bonding catalysis to provide chiral naturally occurring motifs in a straightforward way (a previous synthesis of **12** involving a transition-metal-catalysed hydrogenation to install the stereogenic centre required five steps from a commercially available starting material^9^).

**Scheme 4:**
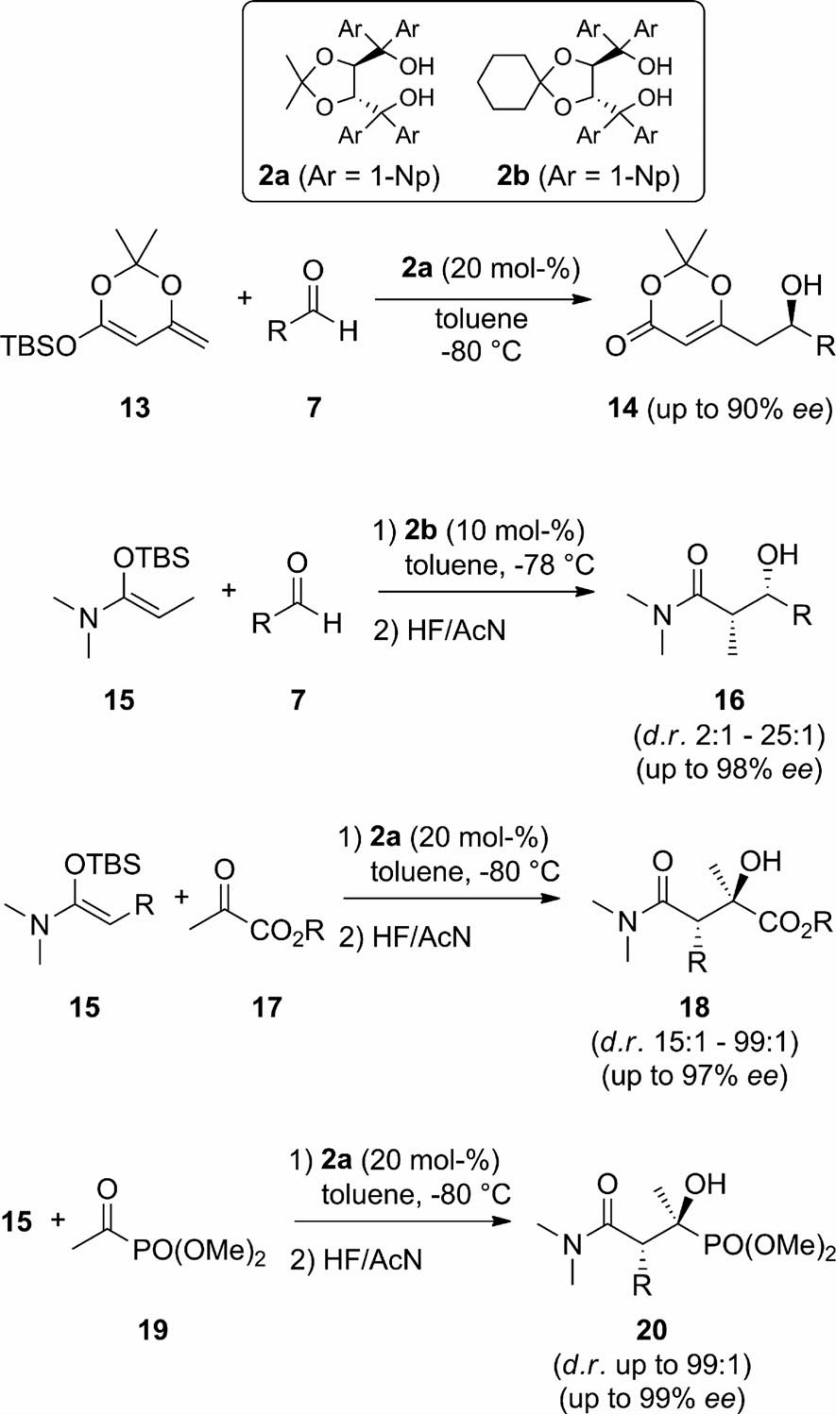
Rawal’s TADDOL-catalysed (vinylogous) Mukaiyama aldol reactions.

The Rawal group has also developed highly stereoselective (vinylogous) Mukaiyama aldol reactions using TADDOLs as readily available H-bonding catalysts (Scheme 4).^10^ Notably, TADDOLs performed significantly better in the vinylogous aldol reactions than other axially chiral diols that were tested (Scheme 4 upper part), not only in terms of enantioselectivity, but also with respect to level of conversion and yield.10a,^11^ Whereas the acetonide-based catalyst **2a** was preferred for the vinylogous addition, the cyclohexanone-ketal-based **2b** performed even slightly better in highly dia- and enantioselective Mukaiyama aldol reactions between *O*-silyl-substituted N,O-acetal **15** and different aromatic (and also one aliphatic) aldehydes **7**.10b In addition, catalyst **2a** was also found to catalyse additions of differently substituted silylated enolates **15** both to β-keto esters **17**10c and to acetyl phosphonate **19**10d efficiently, thus illustrating the broad application scope of this methodology (Scheme 4).

**Scheme 5:**
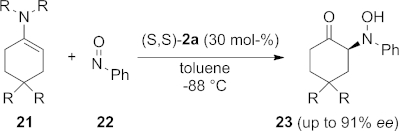
TADDOL-catalysed *N*-nitroso aldol reactions of enamines.

In 2005 Yamamoto et al. investigated Brønsted-acid-catalysed asymmetric nitroso aldol reactions between enamines **21** and nitrosobenzene (**22**, Scheme 5).^12^ Whereas the use of carboxylic acids led to formation of the *O*-nitroso aldol products, use of TADDOL **2a** gave the *N*-nitroso aldol product **23** exclusively and with high enantioselectivity.^12^

**Scheme 6:**
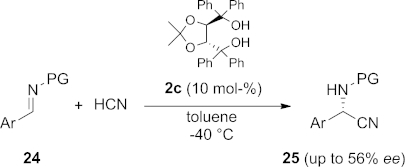
TADDOL-catalysed Strecker reaction.

Another interesting report relating to the potential of TADDOLs as H-bonding catalysts was published in 2007 by Rueping’s group.^13^ During their investigations into organocatalysed enantioselective Strecker reactions they found that BINOL-derived phosphoric acids are versatile catalysts, giving the products in good yields and with high enantioselectivities. In addition, in the course of a thorough screening of different catalysts they also demonstrated the potential of TADDOLs as H-bonding catalysts for the addition of HCN to protected aldimines **24** (Scheme 6). Although the achieved enantioselectivities were only moderate (<56 % *ee*), this report showed that TADDOLs could also be employed for the stereoselective activation of imines.^13^

**Scheme 7:**
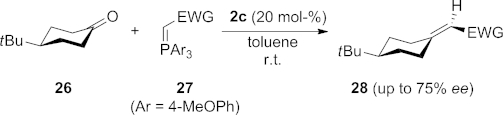
H-bonding-catalysed asymmetric Wittig reactions.

Bernardi’s group recently reported H-bonding-catalysed asymmetric Wittig reactions of 4-substituted cyclohexanones **26** (Scheme 7).^14^ This symmetry-breaking approach gives access to axially chiral olefins **28** in an unprecedented fashion. In the course of these investigations a variety of different chiral H-bonding donors such as ureas, thioureas, sulfonimides, carboxylic acids and diols were screened. Interestingly, TADDOL **2c** was found to be the most efficient catalyst with respect to enantioselectivity (up to 75 % *ee*). The reaction rate was found to be highly dependent on the ylide structure and it was found necessary to use phosphonium ylides **27**, containing electron-rich aryl groups, to achieve good levels of conversion.^14^

**Scheme 8:**
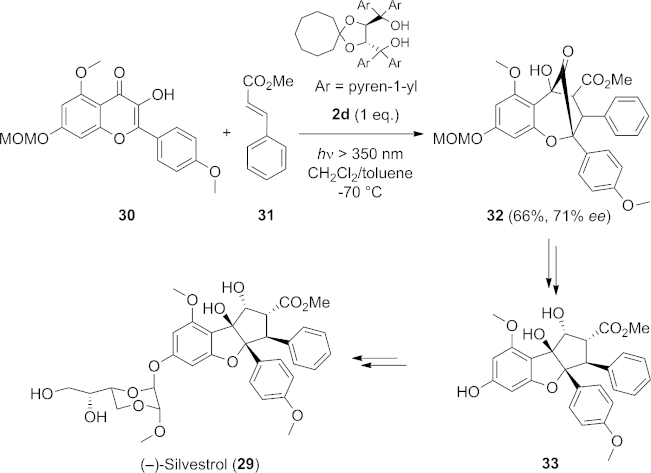
TADDOL-mediated stereoselective photocyclization in the total synthesis of silvestrol (29).

In 2007, Porco’s group reported the total synthesis of the complex rocaglate silvestrol (**29**, Scheme 8), a compound showing very potent cytotoxic activity against human lung cancer cells.^15^ A key step in this synthesis was a photocyclization between the hydroxyflavone **30** and cinnamate **31** by a procedure developed earlier by the same group^16^ in the presence of an equimolar amount of TADDOL **2d**. Although not catalytic in its use of the chiral hydrogen bonding donor, this example highlights the potential of chiral diols (especially TADDOLs) to facilitate complex transformations in a stereoselective manner. Notably, the nature both of the ketal side chain and of the aryl group of the TADDOL are crucial for achieving the cycloadduct **32** with reasonable enantioselectivity. An elegant α-ketol rearrangement and further functional group manipulations then gave the rocaglate derivative **33**, which was finally successfully employed to obtain silvestrol (**29**).^15^ From these impressive examples it is thus undeniable that easily available TADDOLs are a highly powerful and versatile class of chiral H-bonding donor catalysts.^17^

### Phosphoric Acid Derivatives

Chiral phosphoric acids have been amongst the most successfully used chiral Brønsted acids so far.^5^ Interestingly, whereas TADDOL-derived phosphorus-containing ligands have achieved a prominent position in (transition) metal catalysis,^2^,^18^ the backbone of choice for chiral phosphoric acids has so far mainly been the binaphthyl skeleton, with TADDOL-derived phosphoric acids having been used only sparingly in the past. One possible concern in this approach is the fact that the synthesis and handling of a catalyst containing a relatively strong acidic group together with the acid-labile acetal moiety might be a major limitation in relation to other chiral backbones.^19^ However, it has been impressively shown that such TADDOL-phosphoric acids are accessible and have potential for demanding applications.

**Scheme 9:**
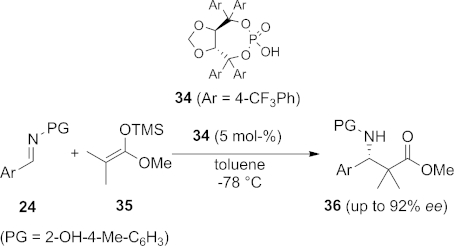
TADDOL-derived phosphoric-acid-catalysed stereoselective Mannich-type reactions.

Akiyama’s group have for years been amongst the frontrunners in asymmetric phosphoric acid catalysis, and in 2005 they also investigated the applicability and potential of TADDOL-derived phosphoric acids for enantioselective Mannich-type reactions (Scheme 9).^20^ Notably, the formaldehyde-based catalyst **34** was found to be the most powerful catalyst, whereas use of other acetal moieties significantly reduced the performance. In addition, the nature of the imine protecting group was found to be crucial for high selectivities; this can be interpreted in terms of a well-defined transition state in which the phosphate hydrogen activates the imine and the phosphoryl oxygen interacts with the OH group of the protecting group. Although this report clearly demonstrated the feasibility of this strategy, and although the corresponding catalysts could be systematically fine-tuned and synthesized in a straightforward fashion, these catalysts have so far been used only to a limited extent. Whereas Charette’s group successfully employed them as ligands in Zn-mediated asymmetric cyclopropanation reactions,^21^ List et al. tested them as chiral Brønsted acids in recent investigations into asymmetric spiroacetalization and transacetalization reactions.^22^ In these special cases, however, the TADDOL-derived phosphoric acids only gave racemic products, whereas other skeletons were found to be much more selective.^22^

### Thioureas

**Scheme 10:**
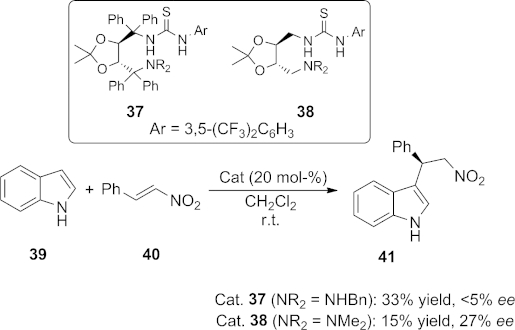
Thiourea-containing catalysts derived from tartaric acid and TADDOLs.

Chiral thioureas are a privileged class of catalysts for noncovalent stereoselective activation of different starting materials.^5^ Surprisingly, though, investigations into the use of thioureas derived from tartaric acid or TADDOLs have so far been rare. At the beginning of 2012, Gherase et al. investigated the synthesis of TADDOL-derived bis(thio)ureas and their carboxylate complexation potential,^23^ but no application as asymmetric organocatalysts was reported. Just recently, Paradies et al. investigated the syntheses of a variety of differently substituted bifunctional thioureas **37** and **38** (Scheme 10), derived from tartaric acid and TADDOLs, and applied them for the Friedel–Crafts alkylation of indole (**39**).^24^ Unfortunately, the catalyst turnovers of these thioureas at room temperature were found to be rather low, and only low levels of enantioselectivity have been achieved so far.^24^ One of the main problems with these catalysts seems to be intramolecular H-bonding between the thiourea and the amino functionality,^23^,^24^ thus resulting in only poor substrate coordination. Accordingly, in view of the results reported so far, the tartaric-acid-derived *trans*-dioxolane-containing moiety seems to be less suited for bifunctional thiourea-containing organocatalysts.

## Chiral Phase-Transfer Catalysts

The design, syntheses, and applications of chiral phase-transfer catalysts (PTCs) have attracted considerable interest over the last three decades.^25^ The great potential of asymmetric phase-transfer catalysis can be attributed to several factors (e.g., mild aqueous reaction conditions, operational simplicity, easily handled catalysts, scalability, …), making it a powerful and versatile methodology for a broad scope of different applications. Besides the use of quaternary ammonium salts based on cinchona alkaloids and Maruoka’s binaphthyl designer catalysts, tartaric acid has proved its potential as a readily modifiable and useful chiral starting material for production of powerful PTCs.

### TADDOLates

**Scheme 11:**
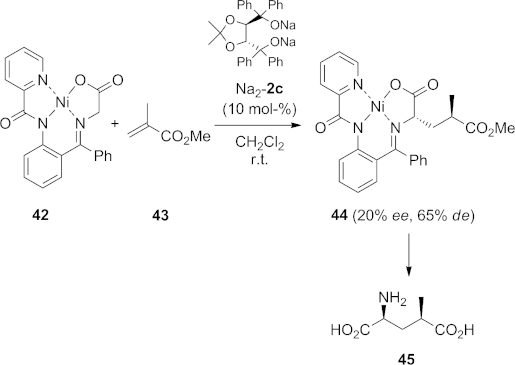
Initial report relating to TADDOLate-catalysed Michael addition of Ni complex 42.

In 1997 Belokon et al. first reported the use of TADDOLates as efficient solid–liquid PTCs.^26^ In this approach, a compound of type **2** acts as a chiral base upon in situ deprotonation with a solid base.^26^,^27^ Initially the asymmetric Michael addition of nickel complex **42** (Scheme 11) to acrylate **43** was investigated. It was found that the disodium salt of **2c** catalysed the reaction to give the Michael product **44** with modest enantio- and diastereoselectivity (20 % *ee* and 65 % *de*). A recrystallization followed by hydrolysis then gave access to enantioenriched glutamic acid **45**.^26^

**Scheme 12:**
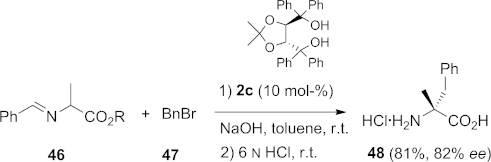
TADDOLate-catalysed asymmetric α-alkylation of alanine Schiff base 46.

On the basis of this initial report, Belokon and Kagan then investigated the TADDOLate-catalysed α-alkylation of alanine Schiff base **46** (Scheme 12).^27^ On treatment of starting material **46** with benzyl bromide (**47**) in the presence of catalytic amounts of TADDOL **2c** and NaOH as the base, followed by subsequent imine hydrolysis, the non-natural amino acid **48** could be obtained in high yield and with good enantioselectivity.^27^ The catalytic cycle is believed to proceed via the monodeprotonated Na-TADDOLate, which forms a complex with the enolate of **46**, thus ensuring good face selectivity in the alkylation step.

**Scheme 13:**
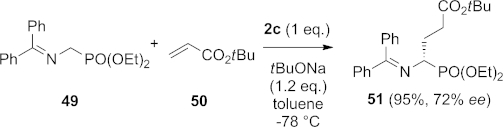
TADDOLate-mediated asymmetric Michael addition of aminophosphonate 49.

TADDOLates were also successfully employed for the asymmetric phase-transfer catalysed Michael addition of aminophosphonate **49** to acrylate **50** (Scheme 13) as shown by Jaszay et al.^28^ After testing of a variety of different catalysts and conditions, the standard TADDOL **2c** in combination with *t*BuONa as the base was identified as the best-suited chiral base, affording the product **51** in high yield and with reasonable enantioselectivity under cryogenic conditions. Unfortunately, the use of stoichiometric amounts of TADDOL was necessary to ensure high selectivities, with use of a catalytic amount only giving almost racemic product.^28^

**Scheme 14:**
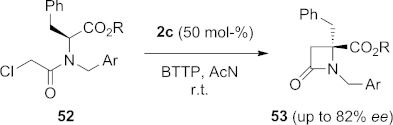
TADDOLate-mediated asymmetric intramolecular alkylation.

An interesting observation relating to the potential of TADDOLates as chiral memory relays was made by Gonzalez-Muniz’s group in their investigations into the stereoselective synthesis of amino-acid-derived β-lactams.^29^ By carrying out intramolecular alkylation of the enantiopure phenylalanine derivative **52** (Scheme 14) in the presence of an achiral base such as BTPP [*tert*-butylimino-tri(pyrrolidino)phosphorane] the lactam **53** could be obtained in around 50 % *ee*. Upon addition of classical cinchona-alkaloid-based PTCs no *ee* enhancement was observed. On addition of a TADDOL, however, the enantiomeric excess could be increased up to 82 % *ee* (with 50 mol-% **2c**). Interestingly, the absolute configuration of the product is determined by the absolute configuration of the starting material rather than that of the chiral catalyst: use of (*S*)-**52** gave (*S*)-**53**, whereas use of (*R*)-**52** instead gave (*R*)-**53**.^29^

### Quaternary Ammonium Salts

Although chiral alkoxides have proved their potential as chiral phase-transfer catalysts, it is fair to say that the majority of described asymmetric PTCs currently in use are based on chiral quaternary ammonium salts.^25^ The use of tartaric acid (**1**) for the production of asymmetric PTCs was pioneered in 2002 by the groups of Shibasaki^30^ and of Arai and Nishida.^31^

**Scheme 15:**
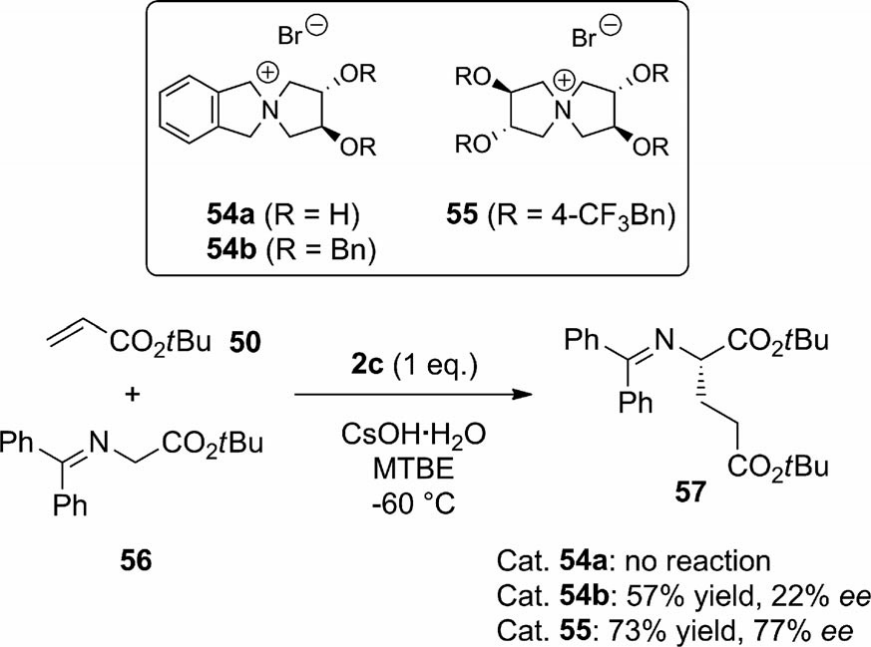
N-Spiro tartaric-acid-derived quaternary ammonium salt PTCs and their use in asymmetric Michael addition reactions.

Arai and Nishida introduced the tartrate-derived *N*-spiro-ammonium salt catalysts **54** and **55** (Scheme 15), which were tested in the asymmetric Michael addition of glycine Schiff base **56** to acrylate **50** under liquid/solid-base phase-transfer conditions.^31^ Whereas the free OH-containing catalyst **54a** did not promote the reaction, the *O*-benzylated **54b** gave the product with low enantioselectivity. In contrast, use of catalyst **55**, containing two tartrate moieties, afforded significantly better selectivities.

Surprisingly, although promising initial results were obtained with catalyst **55**, this catalyst system was to the best of our knowledge not investigated further afterwards.

**Scheme 16:**
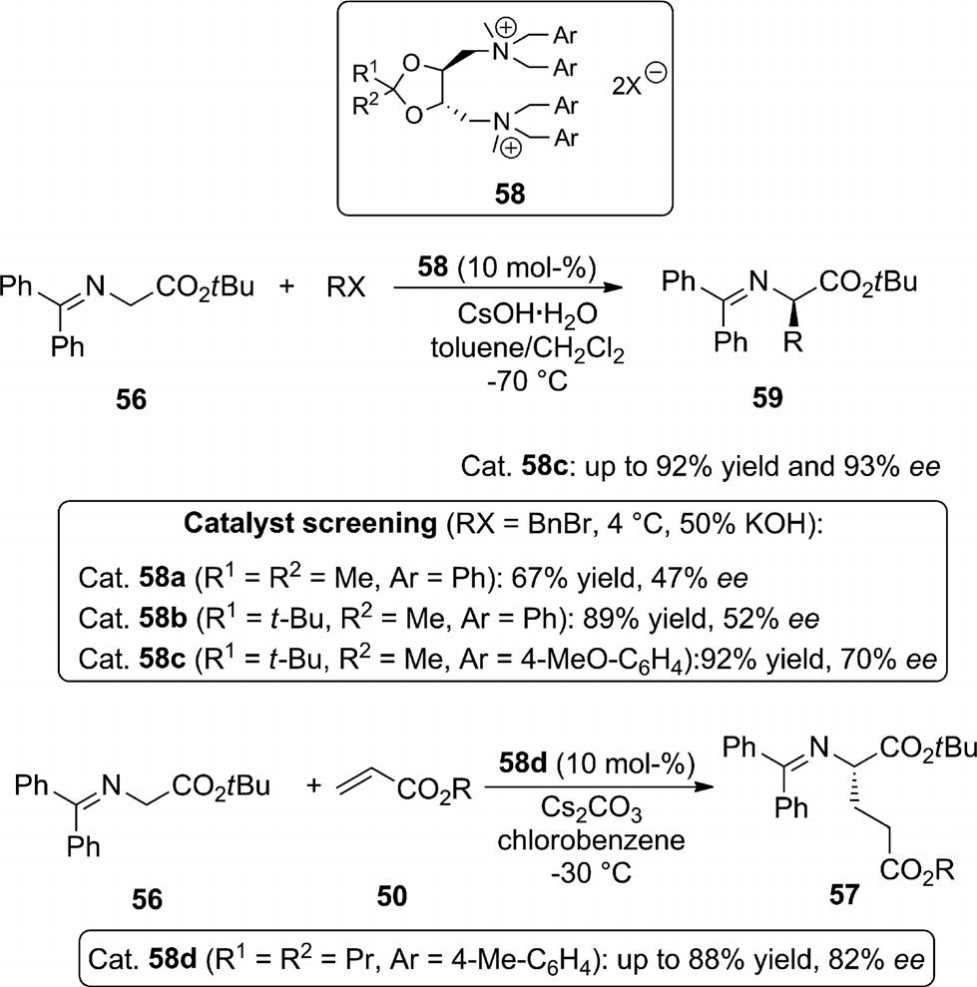
Shibasaki’s tartaric-acid-derived two-centre PTCs.

Inspired by the potential of metal-based two-centre catalysts, Shibasaki et al. introduced a new class of highly active tartaric-acid-derived two-centre PTCs **58** (Scheme 16) in 2002.^30^ The main idea behind this concept was to ensure ideal complexation and orientation of a prochiral nucleophile (e.g., an enolate) by the bidentate catalyst. Tartaric acid (**1**) represents a well-suited starting point here because the developed syntheses allowed for systematic variation either of the ketal groups or of the residues around the quaternary ammonium group, so these catalysts can easily be fine-tuned for a target reaction. In their initial studies, the use of different tartrate-derived diammonium salt catalysts (TaDiAS) for asymmetric α-alkylation and Michael addition reactions of Schiff base **56** was systematically investigated.^30^ Initial findings showed that catalysts with two benzylic groups and a methyl group on the ammonium moiety (compounds **58**) were significantly more active than those containing just alkyl groups or those with just one benzylic group. Notably, whereas *C*_2_-symmetric catalysts such as **58d** were found to be powerful for the Michael addition of **56** to acrylates **50**, *C*_1_-symmetric catalysts were found to be superior for the α-alkylation of **56** (Scheme 16).

On the basis of this pioneering report, Shibasaki’s group then systematically investigated the potential of these catalysts (also by computational means) and their application scope and also applied them for demanding syntheses of biologically active (natural) products.^32^ In the course of these investigations it was also shown that the presence of both ammonium groups was crucial to ensure high selectivities; catalysts containing only one ammonium group performed with only very poor selectivity. In addition, the necessity of the dioxolane moiety was also established, because a corresponding dimethoxy-derived catalyst performed with only low selectivity.^32^ A further advantage of this catalyst system is the easy recoverability of the catalyst afterwards.32c

**Scheme 17:**
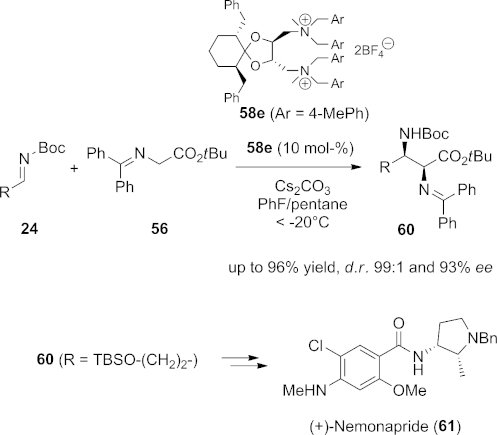
TaDiAS-catalysed Mannich-type reactions.

In addition to further investigations into alkylation and Michael addition reactions, stereoselective Mannich-type reactions have also been carried out in the presence of these catalysts.32d,32f After a thorough screening of different TaDiAS **58** for reactions between glycine Schiff base **56** and Boc-protected imines **24** it was found beneficial to introduce a 2,6-disubstituted cyclohexane-based ketal group and to employ tetrafluoroborates as the counter anions (catalyst **58e**, Scheme 17) to afford the Mannich products **60** with high enantio- and diastereoselectivity even when using enolizable aliphatic imines.32d,32f This Mannich strategy was also successfully employed in a short total synthesis of the antipsychotic agent (+)-nemonapride (**61**).32f

**Scheme 18:**
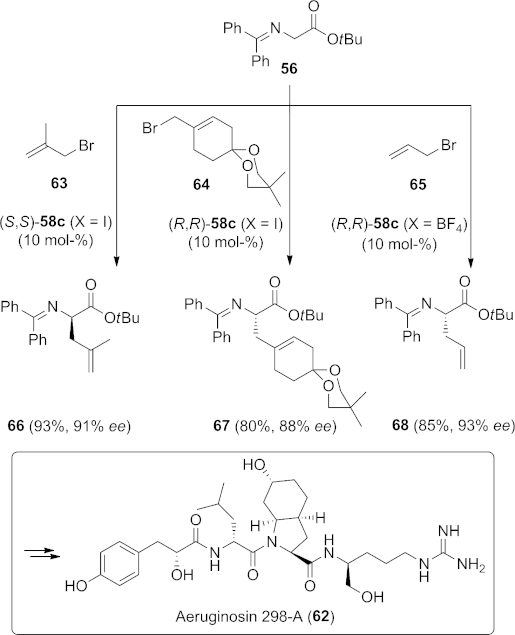
Application of *C*_1_-symmetric TaDiAS 58c in the synthesis of aeruginosin 298-A (62).

As already mentioned, TaDiAS have also been used as chiral catalysts in complex total syntheses. In 2003, Shibasaki’s group reported an elegant synthesis of aeruginosin 298-A (**62**, Scheme 18), a potent serine protease inhibitor isolated from a blue-green alga, using chiral phase-transfer-catalysed alkylations in the syntheses of three fragments.32a,32b Aeruginosin 298-A has a tetrapeptide-like structure including nonstandard amino acids, thus presenting an appropriate motif for phase-transfer-catalysed amino acid syntheses. As depicted in Scheme 18, the two-centre tartaric-acid-derived catalyst **58c** worked well for the installation of the stereogenic centres of the amino acid fragments **66**, **67** and **68** by appropriate alkylation of Schiff base **56** with the corresponding electrophiles in the presence of the matching enantiomer of the *C*_1_-symmetric catalyst **58c**. One interesting fact concerning this methodology is the importance of the catalyst counter anion X^–^ in these alkylation reactions. Whereas catalyst **58c** with X^–^ = I^–^ gave fragment **68** in 79 % yield and 91 % *ee*, the use of BF_4_^–^ as the counter anion gave an even better yield (85 %) and enhanced the enantioselectivity slightly (93 % *ee*). These amino-acid-based fragments could then be assembled successfully to afford aeruginosin 298-A (**62**) in a short and high-yielding sequence.

**Scheme 19:**
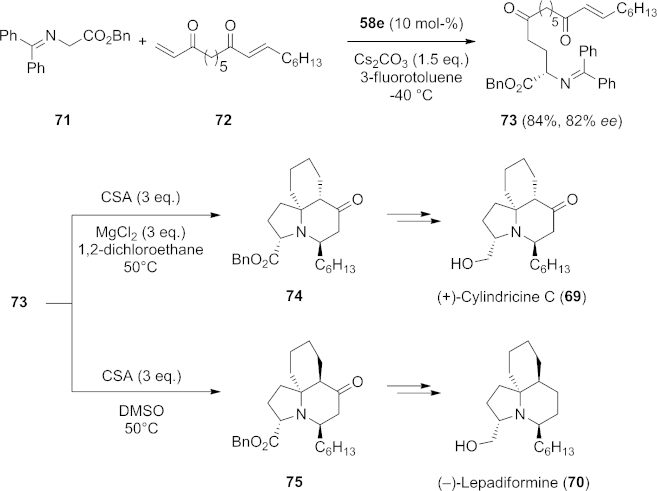
Application of *C*_2_-symmetric TaDiAS 58e in the syntheses of the alkaloids (+)-cylindricine C (69) and (–)-lepadiformine (70).

Shibasaki et al. also demonstrated the applicability of their *C*_2_-symmetric diammonium salt catalyst **58e** for the syntheses of the alkaloids (+)-cylindricine C (**69**, Scheme 19) and (–)-lepadiformine (**70**).32f By applying a **58e**-catalysed addition of Schiff base **71** to Michael acceptor **72**, the key intermediate **73** was obtained in good yield and with a reasonable enantiomeric excess. Compound **73** could then be used to obtain either the cylindricine C precursor **74** or the lepadiformine synthon **75** in selective fashion in very efficient tandem cyclization reactions by appropriate choice of reagents (Scheme 19). The impressively short total synthesis of (+)-cylindricine C (**69**) could be achieved in only two additional steps, whereas the synthesis of the tricyclic intermediate **75** represents a formal total synthesis of (–)-lepadiformine (**70**).32f

**Scheme 20:**
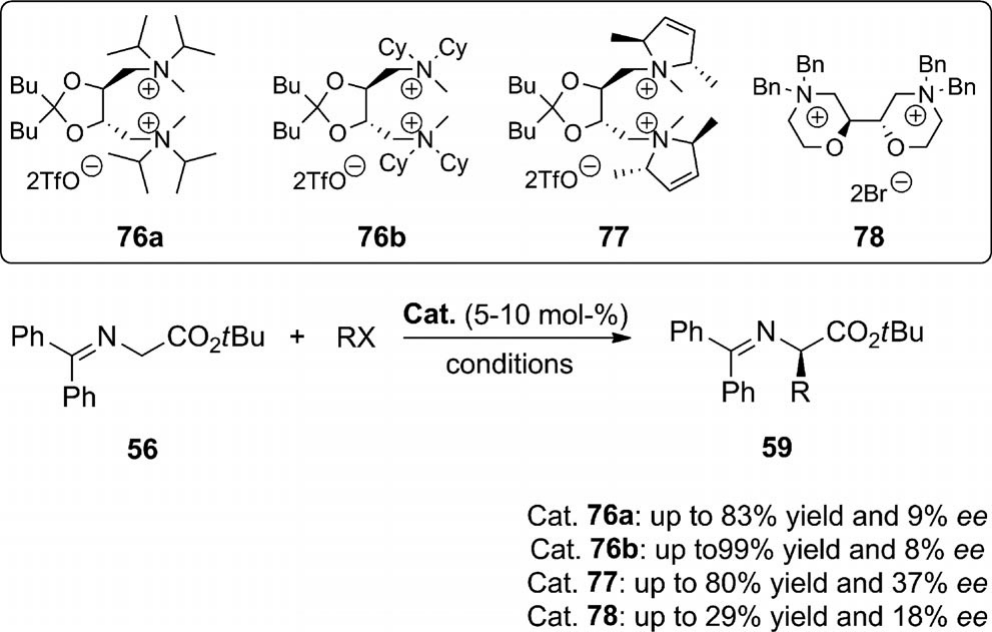
Application of other tartaric-acid-derived two-centre quaternary ammonium salt PTCs.

Besides the attempts of Shibasaki’s group to develop tartaric-acid-derived two-centre quaternary ammonium salt PTCs, MacFarland’s^33^ and Kanger’s^34^ groups also investigated the synthesis and application of such catalysts. MacFarland et al. synthesized and investigated the TaDiAS-related catalysts **76** and **77** (Scheme 20). However, as already observed by Shibasaki et al., tetraalkyl-based 1,4-diammonium salts such as **76** only give low selectivities in asymmetric α-alkylation reactions or Michael additions of Schiff base **56**. The catalytic potential could be slightly improved by incorporating an additional chiral element, as shown in the use of catalyst **77** (Scheme 20).^33^ However, selectivity was still low in comparison with the impressive results obtained with Shibasaki’s catalysts.^32^ Kanger et al. investigated the tartaric-acid-derived bimorpholinium catalyst **78** for the asymmetric alkylation of **56**,^34^ but the yields and enantioselectivities observed with this catalyst system were only modest.

**Scheme 21:**
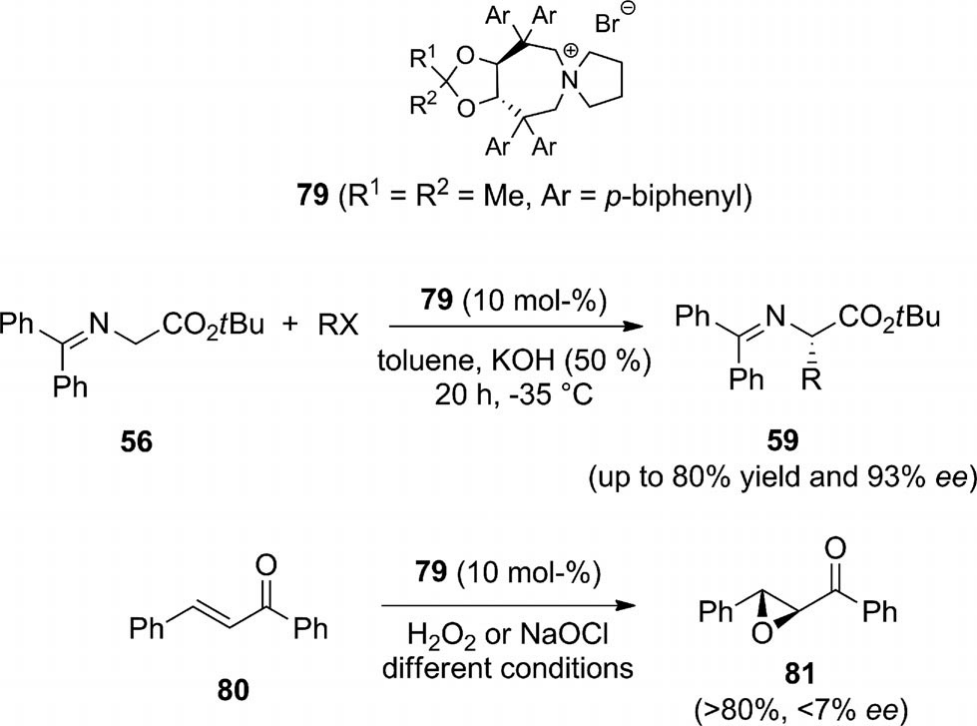
Application of TADDOL-derived *N*-spiro-quaternary ammonium salt PTCs.

Surprisingly, whereas the applicability of tartaric acid (**1**) to provide new ammonium salt PTCs has been thoroughly investigated in the past, syntheses and applications of chiral quaternary ammonium salt catalysts based on TADDOLs **2** have only recently been reported, by our group.^35^ After a careful screening of a variety of differently modified *C*_1_- or *C*_2_-symmetric *N*-spiro catalysts, the *p*-biphenyl-containing acetonide-based catalyst **79** (Scheme 21) turned out to be the most powerful for the asymmetric α-alkylation of **56**. In contrast, testing of this catalyst for the asymmetric epoxidation of chalcone (**80**) resulted only in the formation of racemic **81**. This catalyst motif thus works fine mainly for the activation and control of prochiral nucleophiles, whereas prochiral electrophiles are less selectively activated.^35^

### Chiral Guanidine-Based Catalysts

**Scheme 22:**
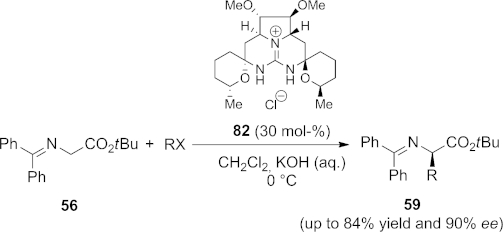
Tartaric-acid-derived chiral pentacyclic guanidine catalyst 82 and its application in the α-alkylation of 56.

Chiral guanidine systems are another promising class of Lewis acidic (phase-transfer) catalysts.^36^–^38^ In 2002 Nagasawa et al. reported the use of the tartaric-acid-derived *C*_2_-symmetric pentacyclic guanidine derivative **82** (Scheme 22) as a powerful chiral catalyst for the benchmark alkylation of Schiff base **56**.^36^ Thorough investigations of differently substituted catalysts revealed that the absolute configuration of the alkylated product **59** is controlled by the configuration of the spiro ether rings of the pentacyclic guanidine core structure of catalyst **82**. In addition, the methyl substituents on the spiro ether rings play a critical role; a similar catalyst lacking these groups only gave almost racemic product.^36^

## Chiral (Lewis) Bases

The use of chiral (Lewis) bases has emerged as one of the most powerful organocatalytic tools for the activation and control of small organic molecules in highly stereoselective applications.^1^,^39^ The scope of application of chiral (Lewis) bases is of course a broad one, ranging from their use as stoichiometric bases through the utilisation of chiral amines in enamine- or iminium-type catalysis to the nucleophilic activation of α,β-unsaturated Michael acceptors in Baylis–Hillman-type reactions and the activation of organosilicon nucleophiles in a variety of different applications.^1^,^39^ Surprisingly, the successful use of **1** or **2** to obtain powerful catalytically active chiral (Lewis) bases has so far been reported only sparingly.^40^

### Chiral Amines

**Scheme 23:**
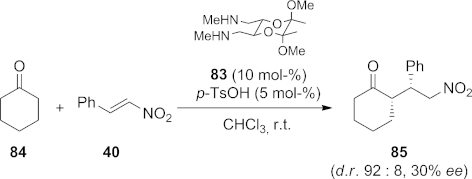
Tartaric-acid-derived diamine 83 in the asymmetric Michael addition of 84 to 40.

Tartaric-acid- or TADDOL-derived amines have been thoroughly investigated as ligands in metal-catalysed reactions,^3^ but their use in organocatalytic reactions has so far attracted only limited interest. In 2006 Barros et al. investigated the use of the tartaric-acid-derived diamine **83** (Scheme 23) for the (dia)stereoselective Michael addition of cyclohexanone (**84**) to Michael acceptor **40**.^41^ The catalyst combination of **83** and *p*-toluenesulfonic acid promoted the reaction efficiently, albeit with only low enantioselectivity.

**Scheme 24:**
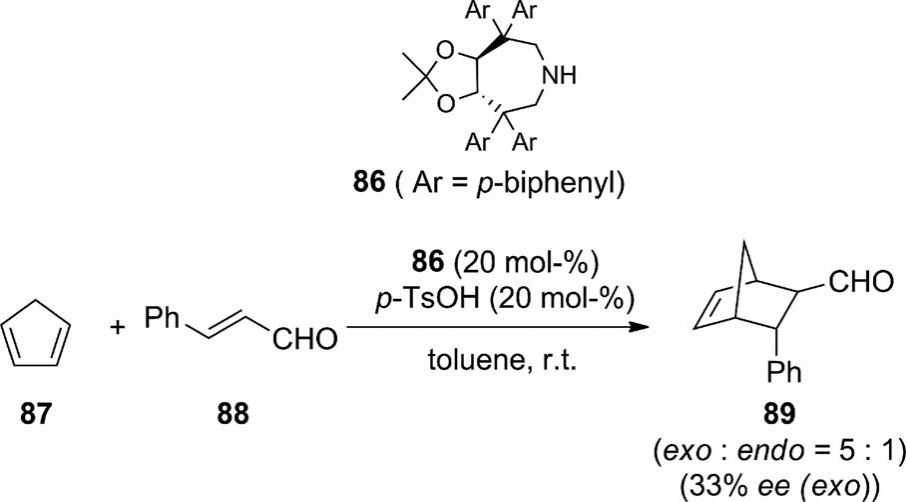
TADDOL-derived secondary amine 86 and its potential for stereoselective Diels–Alder reactions.

During our own investigations into the synthesis of tartaric-acid-based chiral phase-transfer catalysts **79** we synthesized the chiral secondary amine **86** (Scheme 24). On testing this compound for different enamine- or iminium-catalysed reactions we found that it only held promise for iminium activation. Whereas it was not able to catalyse enamine-activated aldol reactions, **86** promoted the Diels–Alder reaction between cyclopentadiene (**87**) and cinnamaldehyde (**88**) well, although with only low enantioselectivity.^42^

### Chiral Formamides and Phosphoramides

**Scheme 25:**
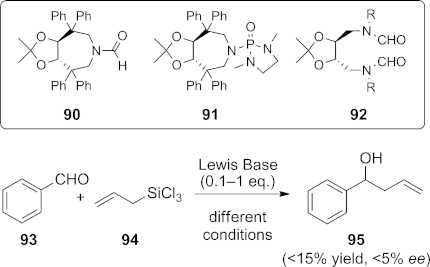
Attempted application of tartaric-acid- and TADDOL-derived chiral formamides and phosphoramides for asymmetric allylation reactions.

Chiral Lewis bases such as formamides or phosphoramides have proved to be useful catalysts for the activation of Lewis acids such as organosilicon nucleophiles.^39^ As part of our own group’s recent research into the development of new tartaric-acid-derived organocatalysts we have recently synthesized the TADDOL-derived formamide **90** (Scheme 25) and the phosphoramide **91**,35b as well as the bidentate tartaric-acid-based bisformamide **92**,^42^ and have tested these compounds in the asymmetric allylation of benzaldehyde (**93**). Unfortunately, though, none of the catalysts promoted the reaction well, even when stoichiometric amounts were used, and the traces of homoallylic alcohol **95** that were isolated showed no optical purity at all, thus making this chiral skeleton unhelpful for chiral formamide and phosphoramide Lewis base catalysts.

### Chiral Phosphites

**Scheme 26:**
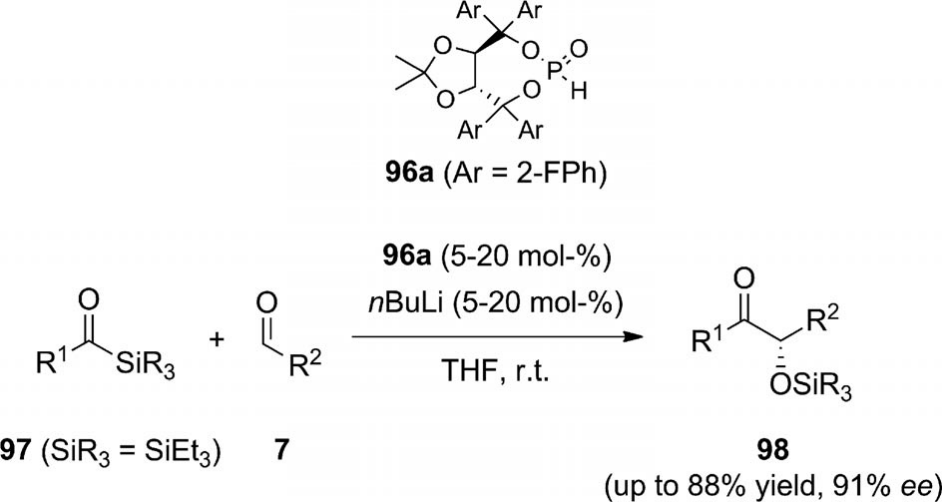
TADDOL-derived metallophosphites as efficient umpolung catalysts for the asymmetric silyl benzoin reaction.

Umpolung strategies are versatile tools for accessing important structural motives. In 2004 Johnson et al. reported the use of TADDOL-derived phosphites **96** (Scheme 26) as efficient umpolung catalysts for the enantioselective cross silyl benzoin reaction.^43^ Upon in situ deprotonation of phosphite **96a** with *n*BuLi, the catalytically active chiral metallophosphite is formed. It then facilitates cross silyl benzoin reactions between acyltriethylsilanes of type **97** and different aldehydes **7** in high yields and with good enantioselectivities for a broad scope of differently substituted aromatic and aliphatic starting materials. As so often with use of TADDOL-derived ligands or catalysts, the nature of the aryl residues and that of the ketal groups were found to be crucial to ensure high selectivities and catalyst turnovers. Interestingly, for these specific reactions the use of BINOL-derived phosphites was found to give racemic products only.^43^

**Scheme 27:**
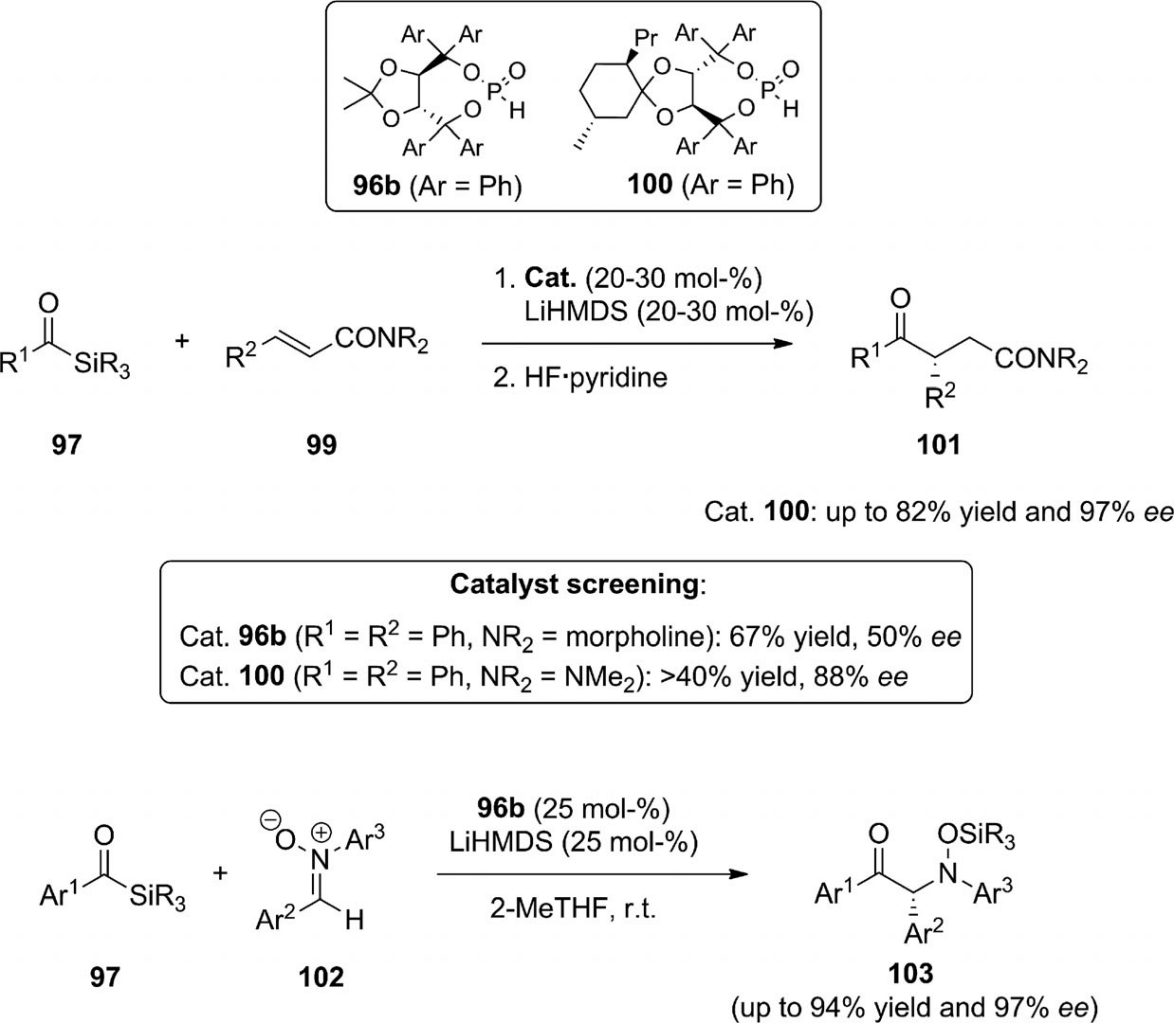
Further applications of Johnson’s umpolung strategy based on TADDOL-based phosphite catalysts.

Johnson’s group later on expanded this methodology to asymmetric acylations of α,β-unsaturated amides and to the C-acylation of nitrones.^44^ Notably, the addition of acylsilanes **97** to α,β-unsaturated amides **99** (Scheme 27) proceeded with moderate enantioselectivity only when the standard TADDOL-derived phosphite **96b** was used.44a Incorporation of an additional element of chirality in the ketal part of the catalyst (compound **100**), however, resulted in a significantly improved enantioselectivity for a broad scope of different Michael acceptors (Scheme 27 – upper reaction).44b In contrast, the enantioselective C-acylation of nitrones **102** could be carried out in good yields and with high selectivities with phosphite catalyst **96b** (Scheme 27 – lower reaction).44c

## Conclusions

Although it is fair to say that tartaric acid or TADDOLs have not achieved such a privileged role as, for example, amino acids or BINOL derivatives as starting materials for the production of new organocatalysts, the reports summarized in this review (and others) have clearly demonstrated the potential of this easily available chiral source for the development of powerful asymmetric organocatalysts. This has been especially impressively demonstrated in the applications of TADDOLs as chiral H-bonding catalysts. In addition to this unique position as one of the most versatile H-bonding motifs currently available, tartrate-derived asymmetric quaternary ammonium salt catalysts have also shown their high potential in numerous case studies in the past. Interestingly, apart from the impressive reports by Johnson’s group on the use of TADDOL-derived phosphites in umpolung reactions, the (Lewis) bases or nucleophilic catalysts developed so far have not been as promising as expected. It is thus obvious that significant efforts to expand the use of tartaric acid as a chiral platform to provide new organocatalysts will be necessary. One lesson that has been clearly taught in these recent reports is the fact that seemingly small changes in the catalyst structure can have a dramatic effect on the catalyst performance. Therefore, the need for flexible synthesis routes to afford carefully diversified libraries of catalysts and to screen them in given target reactions is one of the most important issues to be addressed in the future. In our opinion, however, this approach is a worthwhile task because past results have established that their unique chiral skeleton makes these catalysts versatile alternatives, especially in those cases in which other catalyst moieties do not allow high selectivities.

## Figures and Tables

**Figure:**
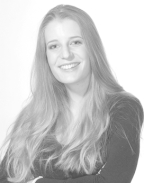


**Figure:**
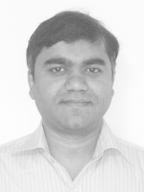


**Figure:**
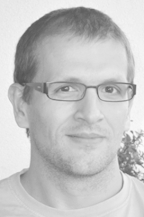


## Biographical Information

Katharina Gratzer was born in Bad Ischl, Austria in 1987. In 2005 she started studying chemistry at the Johannes Kepler University (JKU) Linz, Austria, where she graduated as a Diplom-Ingenieurin in 2011. During her Diploma Thesis in the group of M. Waser she investigated syntheses and applications of TADDOL-derived quaternary ammonium salt catalysts. Since March 2011 she has been working on her PhD thesis in the same group, carrying out ongoing investigations in this field.

Guddeangadi N. Gururaja was born in Sagar, Karnataka, India in 1976 and studied chemistry at IIT Bombay, Mumbai, India, where he obtained his PhD in 2011 in the group of Prof. I. N. N Namboothiri, working on the conjugate addition of bromoform to activated alkenes and studies on bis-homocubyl systems. Since March 2012 he has been undertaking postdoctoral research in the group of M. Waser, where he is working on the syntheses and applications of TADDOL-derived asymmetric organocatalysts.

Mario Waser was born in Steyr, Austria in 1977 and studied chemistry at the Johannes Kepler University Linz, Austria, where he obtained his Ph.D. in 2005 in the group of Prof. Heinz Falk, working on the synthesis of hypericin-based photosensitizers. After postdoctoral work in the group of Prof. Alois Fürstner at the Max-Planck Institut für Kohlenforschung (Mülheim, Germany), where he was part of the team investigating the first total syntheses of iejimalide B and iejimalide A, he spent two years as an R&D chemist for DSM Linz. In 2009 he started his independent career as an Assistant Professor at the JKU Linz. His main research interests are on the design and application of new tartaric-acid-derived organocatalysts, bifunctional phase-transfer catalysts, and ammonium-ylide-mediated reactions.

## References

[1a] Berkessel A, Gröger H (2005). Asymmetric Organocatalysis: From Biomimetic Concepts to Applications in Asymmetric Synthesis.

[b] Dalko I (2007). Enantioselective Organocatalysis.

[c] Waser M, Kinghorn A-D, Falk H, Kobayashi J (2012). Prog. Chem. Org. Nat. Prod..

[2a] de Maria PD (2010). ChemCat-Chem.

[b] Lacoste E, Vaique E, Berlande M, Pianet I, Vincent J-M, Landais Y (2007). Eur. J. Org. Chem..

[c] Czarnecki P, Plutecka A, Gawronski J, Kacprzak K (2011). Green Chem..

[d] Gangwar N, Kasana VK (2011). Synth. Commun..

[3a] Seebach D, Keck AB, Heckel A (2001). Angew. Chem..

[b] Pellissier H (2008). Tetrahedron.

[4a] Seebach D, Beck AK, Hayakawa M, Jaeschke G, Kühnle FNM, Nageli I, Pinkerton AB, Rheiner PB, Duthaler RO, Rothe PM, Weigand W, Wünsch R, Dick S, Nesper R, Wörle M, Gramlich V (1997). Bull. Soc. Chim. Fr..

[b] Seebach D, Hayakawa M, Sakaki J, Schweizer WB (1993). Tetrahedron.

[c] Beck AK, Bastani B, Plattner DA, Petter W, Seebach D, Braunschweiger H, Gysi P, Lavecchia L (1991). Chimia.

[d] Seebach D, Pichota A, Beck AK, Pinkerton AB, Litz T, Karjalainen J, Gramlich V (1999). Org. Lett..

[e] Pichota A, Gramlich V, Beck AK, Seebach D (2012). Helv. Chim. Acta.

[f] Pichota A, Gramlich V, Bichsel H-U, Styner T, Knöpfel T, Wünsch R, Hintermann T, Schweizer WB, Beck AK, Seebach D (2012). Helv. Chim. Acta.

[g] Seebach D, Beck AK, Bichsel H-U, Pichota A, Sparr C, Wünsch R, Schweizer WB (2012). Helv. Chim. Acta.

[5a] Doyle AG, Jacobsen EN (2007). Chem. Rev..

[b] Taylor MS, Jacobsen EN (2006). Angew. Chem..

[c] Akiyama T, Itoh J, Fuchibe K (2006). Adv. Synth. Catal..

[d] Cheon CH, Yamamoto H (2011). Chem. Commun..

[e] Rueping M, Kuenkel A, Atodiresei I (2011). Chem. Soc. Rev..

[6] Tohma H, Takizawa S, Watanabe H, Fukuoka Y, Maegawa T, Kita Y (1999). J. Org. Chem.

[7a] Huang Y, Unni AK, Thadani AN, Rawal VH (2003). Nature.

[b] Thadani AN, Stankovic AR, Rawal VH (2004). Proc. Natl. Acad. Sci. USA.

[8] Du H, Zhao D, Ding K (2004). Chem. Eur. J.

[9] Spino C, Mayes N, Desfosses H (1996). Tetrahedron Lett.

[10a] Gondi VB, Gravel M, Rawal VH (2005). Org. Lett..

[b] McFilvra JD, Unni AK, Modi K, Rawal VH (2006). Angew. Chem..

[c] Gondi VB, Hagihara K, Rawal VH (2010). Chem. Commun..

[d] Gondi VB, Hagihara K, Rawal VH (2009). Angew. Chem..

[11] Villano R, Acocella MR, Massa A, Palombi L, Scettri A (2009). Tetrahedron.

[12] Momiyama N, Yamamoto H (2005). J. Am. Chem. Soc.

[13] Rueping M, Sugiono E, Moreth SA (2007). Adv. Synth. Catal.

[14] Gramigna L, Duce S, Filippini G, Fochi M, Franchini MC, Bernardi L (2011). Synlett.

[15] Gerard B, Cencic R, Pelletier J, Porco JA (2007). Angew. Chem.

[16a] Gerard B, Jones G, Porco JA (2004). J. Am. Chem. Soc..

[b] Gerard B, Sangji S, O’Leary DJ, Porco JA (2006). J. Am. Chem. Soc..

[17] Belokon YN, Gugkaeva ZT, Maleev VI, Moskalenko MA, North M, Tsaloev AT (2010). Tetrahedron: Asymmetry.

[18] Lam HW (2011). Synthesis.

[19] Waser M, Haunschmidt M, Himmelsbach M (2010). Monatsh. Chem.

[20] Akiyama T, Saitoh Y, Morita H, Fuchibe K (2005). Adv. Synth. Catal.

[21] Voituriez A, Charette AB (2006). Adv. Synth. Catal.

[22a] Coric I, Müller S, List B (2010). J. Am. Chem. Soc..

[b] Coric I, List B (2012). Nature.

[23] Gherase D, Roussel C (2012). Cent. Eur. J. Chem.

[24] Lauber M, Fröhlich R, Paradies J (2012). Synthesis.

[25a] Maruoka K (2008). Asymmetric Phase Transfer Catalysis.

[b] Shioiri T, Sasson Y, Neumann R (1997). Handbook of Phase-Transfer Catalysis.

[c] O’Donnell MJ, Ojima I (2000). Catalytic Asymmetric Syntheses.

[d] Maruoka K, Ooi T (2003). Chem. Rev..

[e] O’Donnell MJ (2004). Acc. Chem. Res..

[f] Lygo B, Andrews BI (2004). Acc. Chem. Res..

[g] Hashimoto T, Maruoka K (2007). Chem. Rev..

[h] Ooi T, Maruoka K (2007). Angew. Chem..

[i] Novacek J, Waser M (2013). Eur. J. Org. Chem..

[j] Shirakawa S, Maruoka K (2013). Angew. Chem. Int. Ed..

[26] Belokon YN, Kochetkov KA, Churkina TD, Ikonnikov NS, Orlova SA, Smirnov VV, Chesnokov AA (1997). Mendeleev Commun.

[27a] Belokon YN, Kochetkov KA, Churkina TD, Ikonnikov NS, Chesnokov AA, Larionov OV, Parmar VS, Kumar R, Kagan HB (1998). Tetrahedron: Asymmetry.

[b] Belokon YN, Kochetkov KA, Churkina TD, Ikonnikov NS, Chesnokov AA, Larionov OV, Singh I, Parmar VS, Vyskocil S, Kagan HB (2000). J. Org. Chem..

[28] Jaszay ZM, Nemeth G, Pham TS, Petnehazy I, Grün A, Töke L (2005). Tetrahedron: Asymmetry.

[29] Bonache MA, Lopez P, Martin-Martinez M, Garcia-Lopez MT, Cativiela C, Gonzalez-Muniz R (2006). Tetrahedron.

[30] Shibuguchi T, Fukuta Y, Akachi Y, Sekine A, Ohshima T, Shibasaki M (2002). Tetrahedron Lett.

[31] Arai S, Tsuji R, Nishida A (2002). Tetrahedron Lett.

[32a] Ohshima T, Gnanadesikan V, Shibuguchi T, Fukuta Y, Nemoto T, Shibasaki M (2003). J. Am. Chem. Soc..

[b] Fukuta Y, Ohshima T, Gnanadesikan V, Shibuguchi T, Nemoto T, Kisugi T, Okino T, Shibasaki M (2004). Proc. Natl. Acad. Sci. USA.

[c] Ohshima T, Shibuguchi T, Fukuta Y, Shibasaki M (2004). Tetrahedron.

[d] Okada A, Shibuguchi T, Ohshima T, Masu H, Yamaguchi K, Shibasaki M (2005). Angew. Chem..

[e] Shibuguchi T, Mihara H, Kuramochi A, Sakuraba S, Ohshima T, Shibasaki M (2006). Angew. Chem..

[f] Shibuguchi T, Kuramochi A, Ohshima T, Shibasaki M (2007). Chem. Asian J..

[33a] Rueffer ME, Fort LK, MacFarland DK (2004). Tetrahedron: Asymmetry.

[b] Kowtoniuk WE, MacFarland DK, Grover GN (2005). Tetrahedron Lett..

[34] Lippur K, Kanger T, Kriis K, Kailas T, Müürisepp A-M, Pehk T, Loop M (2007). Tetrahedron: Asymmetry.

[35a] Waser M, Gratzer K, Herchl R, Müller N (2012). Org. Biomol. Chem..

[b] Gratzer K, Waser M (2012). Synthesis.

[c] Herchl R, Waser M (2013). Tetrahedron Lett..

[d] Gururaja GN, Herchl R, Pichler A, Gratzer K, Waser M (2013). Molecules.

[36] Kita T, Georgieva A, Hashimoto Y, Nakata T, Nagasawa K (2002). Angew. Chem.

[37a] Nagasawa K, Georgieva A, Takahashi H, Nakata T (2001). Tetrahedron.

[b] Allingham MT, Howard-Jones A, Murphy PJ, Thomas DA, Caulkett PWR (2003). Tetrahedron Lett..

[c] Sohtome Y, Hashimoto Y, Nagasawa K (2005). Adv. Synth. Catal..

[d] Cho B, Tan C-H, Wong MW (2012). J. Org. Chem..

[38] Sereda O, Clemens N, Heckel T, Wilhelm R (2012). Beilstein J. Org. Chem.

[39a] Denmark SE, Beutner GL (2008). Angew. Chem..

[b] Denmark SE, Stavenger RA (2000). Acc. Chem. Res..

[40] Catel D, Chevallier F, Mongin F, Gros PC (2012). Eur. J. Org. Chem.

[41] Barros MT, Phillips AMF (2006). Molecules.

[42] Waser M

[43] Linghu X, Potnick JR, Johnson JS (2004). J. Am. Chem. Soc.

[44a] Nahm MR, Linghu X, Potnick JR, Yates CM, White PS, Johnson JS (2005). Angew. Chem.

[b] Nahm MR, Potnick JR, White PS, Johnson JS (2006). J. Am. Chem. Soc.

[c] Garrett MR, Tarr JC, Johnson JS (2007). J. Am. Chem. Soc.

